# Correlating mechanical work with energy consumption during gait throughout pregnancy

**DOI:** 10.1186/s12884-015-0744-4

**Published:** 2015-11-20

**Authors:** Zarko Krkeljas, Sarah Johanna Moss

**Affiliations:** Physical Activity, Sport and Recreation Research Focus Area, Private Bag x6001, Internal Box 481, North-West University, Potchefstroom, 2520 South Africa

**Keywords:** Gait, Pregnancy, Energy expenditure, Metabolic energy, Mechanical work

## Abstract

**Background:**

Measures of mechanical work may be useful in evaluating efficiency of walking during pregnancy. Various adaptations in the body during pregnancy lead to altered gait, consequently contributing to the total energy cost of walking. Measures of metabolic energy expenditure may not be reliable for measuring energetic cost of gait during pregnancy as pregnancy results in numerous metabolic changes resulting from foetal development. Therefore, the aim of this study is to determine if mechanical work prediction equations correlate with the metabolic energy cost of gait during pregnancy.

**Methods:**

Thirty-five (35) women (27.5 ± 6.1 years) gave informed consent for participation in the study at different weeks of gestation pregnancy. Gas exchange and gait data were recorded while walking at a fixed self-selected walking speed. External (W_ext_) work was estimated assuming no energy transfer between segments, while internal work (W_int_) assumed energy transfer between segments. Hence total energy of the body (W_tot_) was calculated based on the segmental changes relative to the surrounding, and relative to the centre of mass of the whole body. Equations for mechanical work were correlated with net and gross O_2_ rate, and O_2_ cost.

**Results:**

External, internal and total mechanical energy showed significant positive relationship with gross O_2_ rate (*r* = 0.48, *r* = 0.35; and *r* = 0.49 respectively), and gross O_2_ cost (*r* = 0.42; *r* = 0.70, and *r* = 0.62, respectively). In contrast, external, internal and total mechanical energy had no significant relationship with net O_2_ rate (*r* = 0.19, *r* = 0.24, and *r* = 0.24, respectively). Net O_2_ cost was significant related W_ext_ (*r* = 0.49) W_int_ (*r* = 0.66) and W_tot_ (*r* = 0.62). Energy recovery improved with increase in gait speed.

**Conclusions:**

Measures of mechanical work, when adjusted for resting energy expenditure, and walking speed may be useful in comparing metabolic energy consumption between women during pregnancy, or assessment or gait changes of the same individual throughout pregnancy.

## Background

During pregnancy energy required for walking may increase significantly due to an increase in weight [1–3]. Hence walking, as a common activity of daily living, may contribute to an increase in total energy expenditure during pregnancy. Previous studies indicate high variability in gait during pregnancy due to pregnancy-related physical and physiological changes [4], which may result in an increase in mechanical work. The ability to predict changes in mechanical energy based on pregnancy-related adaptations to gait may provide a better understanding of energy balance during pregnancy.

Current research in pregnancy is largely focused on increased energy demands stemming from foetal development, hormonal changes, and changes in physical activity [2, 5–7]. Any increase in energy expenditure during pregnancy has been attributed largely to an increase in resting metabolic rate (RMR) [2], while any increase in the active energy expenditure (AEE) has been attributed primarily to the mass gain during pregnancy [1–3]. This indicates that no other factors have currently been identified that contribute to the energy cost, and that relative to mass, AEE remains unchanged throughout pregnancy. In addition, difficulties in metabolic analysis of gait during pregnancy stem mostly from the large inter-subject variability in physiological changes resulting from pregnancy [4]. These include changes in dietary-induced thermogenesis, pre-pregnancy malnutrition, or energy cost to synthesize new placental tissue.

However, studies examining gait biomechanics during pregnancy report that walking speed lowers significantly throughout pregnancy [1, 4], a behavioural change indicating that women during pregnancy are more comfortable at lower velocities. At lower velocities vertical excursions of the centre of gravity (COG) decrease, which contributes to the increase in energy expenditure [8–11]. In addition, step width tends to increase with pregnancy, a change consistent with the need for an increased balance, also an emphasis on safety, which is characterized by the change in path of the COG [12–17]. This seems to indicate a trade-off mechanism for gait during pregnancy, where women would choose to walk slower with wider steps that may result in an increase in walking energy expenditure, contributing to AEE. This notion contradicts the inherent nature of energy sparing during pregnancy [1, 18, 19]. Hence, the use of mechanical work measures would permit simplified evaluation of changes in walking patterns during pregnancy that may reduce the energy expenditure in walking during pregnancy. However, measures of mechanical work have an inherent weakness stemming from assumptions of energy transfers, relative metabolic cost of positive and negative work, stored elastic energy, captivation of antagonist muscles, and isometric work [20]. As a result, the use of mechanical power output in clinical populations tends to be equivocal. Furthermore, the application of mechanical power output on self-paced walking during pregnancy is also lacking.

Therefore, the purpose of this study was to examine the ability of total body mechanical power to explain the variability in the metabolic energy cost of self-paced walking during pregnancy in a South African cohort of women.

## Methods

This study is ancillary to a larger Habitual Activity Patterns during Pregnancy (HAPPY)-study that investigated the influence of objectively determined physical activity patterns on various pregnancy parameters. Thirty-five (35) pregnant South African women at different stages of pregnancy, mean age 27 years (S.D. = 6.1) were recruited, by advertisements in the local press, the consulting rooms of local gynaecologists, and a local health clinic in Potchefstroom, North West Province, South Africa. For participation in the study, women had to be healthy, between ages 18 and 40 years, without mental or physical disability, and able to complete the test protocol. Participants were excluded from the study, if they presented with physical limitations that may prevent movement, the inability to complete test procedures, or were considered high-risk pregnancy according to ACSM guidelines [21]. The study was approved by the Human Research Ethics committee of the North-West University (NWU-0044-10-A1). Participants gave written consent for participation in the study before data collection. A translator was available in the case of language barriers. Participants were informed that they are free to withdraw from the study at any point. In addition, at the day of motion analyses testing participants were free to withdraw from this specific protocol. An opportunity to ask questions was also given.

Participants RMR was assessed using the fraction of oxygen in expired gases (Cosmed Fitmate, Italy), applying standard metabolic formulas, while energy expenditure was calculated using a fixed respiratory quotient (RQ) of 0.85 [22]. Before the Fitmate was attached, participants were laying still for 5 min on their left side, to ensure resting state. Prior to gas collection, participants were connected to the Fitmate for no longer than two (2) minutes to ensure that all dead space and any other gases are flushed prior to data collection. Following the initial 2 min preparation period, RMR gas exchange is collected for 16 min per Fitmate RMR protocol. Participants were instructed not to perform any exercise 24 h prior to testing, and fast for at least 10 h prior to the measurement. A calibration of the Fitmate was done before each participant was subjected to a measurement.

Walking energy expenditure was measured using portable K4b^2^ (Cosmed, Italy) system. Prior to each measure, the system was calibrated for O_2_ concentration, gas volume and delay, according to the manufacturer’s recommendations. Participants walked at a self-selected pace along a 30-m oval track in the laboratory until steady state was reached. Steady state was considered by HR variation of being no more than ±3 bpm, and less than 5 % variation of RQ [23], during which RQ of less than ≤ 0.99 has to be maintained (indicative of still using oxidative system, and not reaching fatigue state) [24]. Walking metabolic rate was averaged for the full minute the steady state was reached. Although participants were instructed to rest at any point if they felt tired before reaching steady state, no participant exercised this option. All the parameters were collected at 2-s intervals. The following parameters were extracted: walking volume of oxygen (VO_2_), respiratory quotient (RQ), resting metabolic rate (RMR) (kcal/day), heart rate (HR)(bpm), and gross energy expenditure during walking per minute (GEE_w_).

Three dimensional (3-D) gait analysis was collected using eight Oqus 300+ cameras from Qualisys Motion Analysis System (Qualisys, Sweden) and filmed at 220 Hz. Before each gait data analysis, a 90-s wand (750 mm) calibration was completed, with a long arm of a L-shaped reference structure. Participants were dressed in appropriate clothing, cycling shorts and a tank top which would allow marker placement on the skin and minimize any artefacts from clothing movement. A full-body CAST/IK/HH gait model was used, requiring 12-mm self-adhesive reflective markers to be applied on the following anatomical landmarks (right and left): heel at the insertion of the Achilles tendon, head of the first, second and fifth metatarsal, medial and lateral malleoli, lower leg (shank) cluster consisting of 4 markers, lateral and medial knee epicondyles, thigh cluster consisting of 4 markers, greater trochanter, anterior and posterior superior iliac spine, inferior angle of scapula, thoracic vertebrae (T10), cervical vertebrae (C7), radial and ulnar styloid processes, humeral lateral epicondyle, humerus, acromion. This full marker set was used for a static trial only, which required the participant to stand still for 5 s while filmed in the centre of the calibrated area. Static trials were used to create a dynamic model for gait analysis. Once the static trial was completed, only dynamic markers were left, hence the markers on the medial and lateral malleoli, knee epicondyles, and the trochanter were removed.

During the dynamic trials, each participant was instructed to walk in a straight line at a self-selected pace along a 15-m laboratory walkway embedded with 4 AMTI BP400600 force plates (AMTI, MA, USA). Video and ground reaction force data were collected simultaneously for five seconds in the middle portion of the runway. Only trials in which the participant’s foot landed entirely on a force plate for three consecutive steps (i.e. full stride), were considered for inclusion in the data set. The participants continued walking until 3 trials of full strides were completed. The participants were instructed to stop and rest as long as necessary, should they feel tired at any stage of the gait analyses. None of the participants requested a rest period during gait measures.

### Data analysis

During walking trials, the data were inspected for gaps in marker trajectories. The default gap-fill function was applied for gaps of no more than 10 frames using NURB spline interpolation. No walking data trials analysed had gaps larger than 10 frames. Once the walking trials were trimmed to include completed strides, the data were exported to Visual 3D-motion analysis software for processing, through which segmental and whole-body kinetic data and gait kinematic data were calculated. The kinetic and kinematic parameters were low-pass filtered with a bidirectional Butterworth with a 10-Hz cut-off frequency to remove noise from the differentiation process with zero-phase distortion [13, 25].

Metabolic energy expenditure is generally reported in terms of O_2_ consumption (O_2_ rate), the millilitres of O_2_ consumed, per kilogram body mass per minute (ml/kg/min). However, to demonstrate the physiological work (O_2_ cost) for a given task, the physiological equivalent rate will also be normalized for speed to express the physiological work per unit distance (ml/kg/m) which is used to depict energy efficiency [10]. Therefore steady state walking total O_2_ cost might be affected by an increase in O_2_ rate, or a change in a walking speed, in which case the participant would not experience any physical differences. To reduce the impact of changes in resting metabolic rate (RMR), a non-dimensional parameter (NN) was used as it deduces the resting energy expenditure from gross or total energy expenditure during walking, leaving only energy cost required for walking and non- dimensional scaling to account for stature [26]. The net O_2_ cost may also be less sensitive to changes in walking speed [11]. While this method theoretically accounts for the pregnancy induced changes there are no articles addressing this normalization for gait in pregnancy.

Mechanical work and total energy expenditure were explained in detail in Bennett et al. [16], and Willems et al. [27], hence only a brief summary will be provided in this study. Internal (W_int_) and external work (W_ext_), were calculated from COM excursion considering energy exchange as

1 $$ {\mathrm{W}}_{\mathrm{ext}}={\displaystyle \sum_{i=1}^N\ \left(\left|\varDelta \mathrm{E}\mathrm{p}\right|+\left|\varDelta \mathrm{E}\mathrm{k}\right|\right)}, $$

and with no energy exchange as

2 $$ {\mathrm{W}}_{\mathrm{int}}={\displaystyle \sum_{i=1}^N\ \left(\left|\varDelta \mathrm{E}\mathrm{p}\right|+\left|\varDelta \mathrm{E}\mathrm{k}\right|\right)}, $$

where ∆Ep and ∆Ek are changes in potential and kinetic energy, respectively [16]. Then, the energy recovery factor (R) representing the percentage of mechanical energy recovered via exchange between kinetic and potential energy in the COM movement is computed as [16]:

3 $$ \mathrm{R}=100 \times \frac{\left({\mathrm{W}}_{\mathrm{int}}-{\mathrm{W}}_{\mathrm{ext}}\right)}{{\mathrm{W}}_{\mathrm{int}}} $$

Further, total energy of the whole body based on the segmental movement relative to COMwb at any instant in time as

4 $$ {\mathrm{E}}_{\mathrm{tot},\mathrm{w}\mathrm{b}}=\mathrm{M}\mathrm{g}\mathrm{H}+\frac{1}{2}\mathrm{M}{{\mathrm{V}}_{\mathrm{cg}}}^2+{\displaystyle \sum_{i=1}^N\ \left(\frac{1}{2}{\mathrm{m}}_{\mathrm{i}}{{\mathrm{v}}_{\mathrm{i}}}^2+\frac{1}{2}{\mathrm{m}}_{\mathrm{i}}{{\mathrm{K}}_{\mathrm{i}}}^2{\upomega_{\mathrm{i}}}^2\right)}, $$

where *M* is the total body mass; *g* the acceleration due to gravity; *H* is height of the COM; *V*_*cg*_ the velocity of the COG; *m*_*i*_ and *v*_*i*_ are mass and velocity of the *i*th segment relative to the surrounding; *ω*_*i*_ and *K*_*i*_ are the angular velocity and the radius of gyration of the *i*th segment around its centre of mass [27].

### Statistical analysis

A one-way ANOVA was used to assess differences between trimesters for descriptive participants’ parameters. Simple linear regression was used to calculate the relationship between metabolic energy and mechanical work. All analyses were performed using SPSS v.21.0 (IBM Corp., Armonk, NY), and significance set at *p* <0.05.

## Results

The participant demographics by trimester are given in Table 1. Some of the participants completed analysis at multiple trimesters totalling 44 measures during the total period of pregnancy. There were no differences in age and height between participants in each trimester, although mass gain was significantly increased throughout pregnancy as the foetus grew.

**Table 1:** Descriptive statistics of the participants by trimesters

Measure	Total Mean ± SD	1^st^ trim Mean ± SD	2^nd^ trim Mean ± SD	3^rd^ trim Mean ± SD	Sig. (p)
Participants (n)	35^b^	14	20	10	
Age	27.5 ± 6.1	28.1 ± 5.5	27.1 ± 6.1	26.6 ± 6.6	0.83
Height (cm)	160.8 ± 6.4	160.8 ± 5.9	160.2 ± 6.8	161.4 ± 7.2	0.89
Mass (kg)	70.4 ± 15.7	62.7 ± 10.5	71.3 ± 16.6	78.8 ± 14.7	0.08
BMI (kg/m^2^)	27.3 ± 5.9	24.3 ± 4.0	27.7 ± 6.2	29.9 ± 4.9	0.08
M_gain_ (kg)	6.1 ± 6.3	1.09 ± 3.1	5.27 ± 2.8	13.81 ± 7.9	0.00
PPM (kg)^a^	64.4 ± 14.5				
PPBMI (kg/m^2^)	25.1 ± 5.5				
Ethnicity	74.3 % black				
	17.1 % mixed ancestry				
	8.6 % white				

*M*_*gain*_ mass gain from pre-pregnancy (i.e. total mass gain), *PPM* pre-pregnancy mass; ^*a*^Self-reported; *PPBMI* pre-pregnancy body mass index; trim trimester; ^b^Several participants were measured in multiple stages

**Table 2:** Regression equation for metabolic energy expenditure and mechanical work

	W_ext_ (J/kg/min)	W_int_ (J/kg/min)	W_tot_ (J/kg/min)
Net O_2 cost_ (ml/kg/m)	1.1646O_2_ + 0.7314	0.889O_2_ + 0.2807	2.0536O_2_ + 1.0121
Net O_2__rate_ (ml/kg/min)	0.0045O_2_ + 0.8211	0.0032O_2_ + 0.3514	0.0077O_2_ + 1.1724
Gross O_2 cost_ (ml/kg/m)	0.9865O_2_ + 0.7032	0.9384O_2_ + 0.2299	1.925O_2_ + 0.9331
Gross O_2 rate_ (ml/kg/min)	0.0115O_2_ + 0.742	0.0113O_2_ + 0.2624	0.0228O_2_ + 1.0044

*W*_*ext*_ external mechanical work, *W*_*int*_ internal mechanical work, *W*_*tot*_ total work

**Fig. 1:**
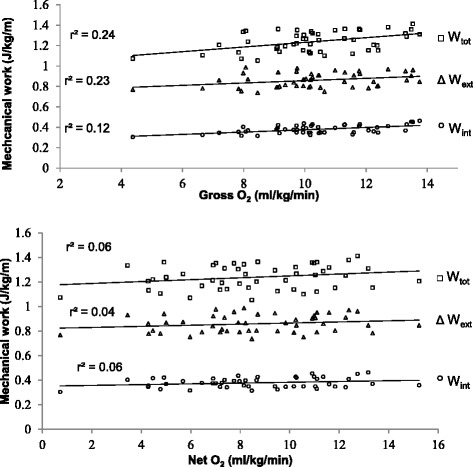
Relationship between metabolic rate and mechanical work of walking during pregnancy

**Fig. 2:**
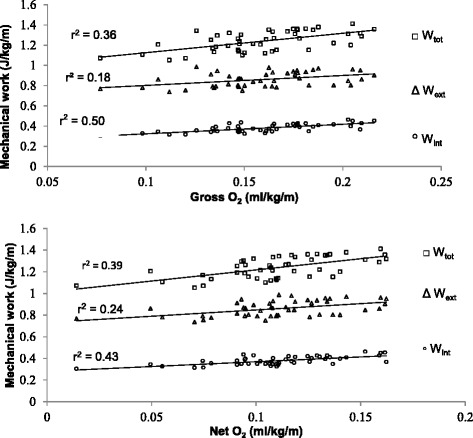
Relationship between metabolic cost and mechanical work of walking during pregnancy

**Fig. 3:**
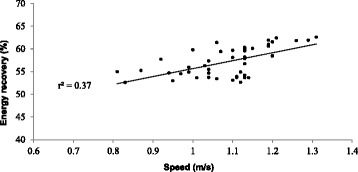
Relationship between mechanical energy recovery and walking speed throughout pregnancy

Prediction equations for external (W_ext_), internal work (W_int_) and total work (W_tot_) depict no significant relationship with net O_2_ expenditure of walking during pregnancy (*p* =0.19, *p* =0.10 and *p* = 0.11, respectively), Fig. 1. However, once normalized for speed to express energy efficiency (net O_2_ cost) (Fig. 2), prediction equations (Table 2) show moderate, but significant relationship for external work (W_ext_) (*r* = 0.49, *p* ≤ 0.01), internal work (W_int_) (*r* = 0.66, *p* ≤ 0.00), and total work (W_tot_) (*r* = 0.63, *p* ≤ 0.01). Although net O_2_ expenditure did not demonstrate a significant relationship to mechanical work, adding REE resulted in a significant relationship to mechanical work. Gross O_2_ expenditure during walking shows a weak to moderate, but significant relationship to W_int_ (*r* = 0.60, *p* ≤ 0.01), moderate with W_ext_ (*r* = 0. 42, *p* ≤ 0.01), as well as with W_tot_ (*r* = .71, *p* ≤ 0.01). When considering REE in walking energy cost, the relationship with mechanical work as demonstrated in Fig. 2, did not change significantly. The mass is also the most significant (*r* = 0.79, *p* ≤ 0.01) contributor in variance of gross O_2_ consumption (VO_2_) with 33.3 %, next to walking speed (10.0 %, *p* ≤ 0.05). However, once normalized for REE, walking speed explained 26.1 % of the net energy expenditure (*p* ≤ 0.01). Energy recovery factor (R) for the pregnant population in this study was 58.1 ± 3.2 %. The exchange of potential and kinetic energies, a determining factor for energy recovery, was largely affected by speed of walking. Figure 3 demonstrates a significant relationship between speed and energy recovery (*r* = 0.61, *p* ≤ 0.01), indicating that during pregnancy energy recovered may be improved with an increase in speed. However, in this study pregnant women decrease their walking speed later in pregnancy. The changes observed are an indication of an increase in walking economy with an increase in walking speed.

## Discussion

This study addressed the relationship between metabolic energy expenditure and mechanical work measures of gait throughout pregnancy. With significant findings in studies on gait in pregnancy, as well as energy expenditure during pregnancy, researchers may be able to determine whether changes in gait during pregnancy may contribute to the overall energy expenditure, and whether changes in gait may be used as an energy-sparing strategy during pregnancy.

The study finds significant relationship between W_int_, W_ext_, and W_tot_, and net O_2_ and gross O_2_ energy cost, and gross O_2_ rate, but not for net O_2_ rate. The significance between parameters, however, was of different magnitudes and was relevant to the resting energy expenditure, and the normalization for the walking speed. The changes in metabolic system due to foetal development will affect the resting energy expenditure, and consequently the total energy expenditure during pregnancy. This change cannot be accounted for by mechanical work measures, and metabolic changes in resting energy expenditure are also generally based on the estimates. In this study normalizing gross O_2_ cost for resting energy expenditure, resulted in a stronger correlation, which suggests that normalization for resting energy expenditure does not decrease the internal consistency of data [23], and reduces the variability of the metabolic changes from foetal development.

Measuring metabolic energy consumption relative to distance or time travelled, may also affect the strength of the correlation [10, 26, 28]. Burdet et al. [28] found that the correlation of mechanical work with metabolic energy consumption per meter walked (ml/kg/m) weaker than when metabolic consumption was measured per time (ml/kg/s). However, Schwartz et al. [26] and Waters and Mulroy [10] demonstrate that normalizing for walking speed would give a better indication of walking efficiency. In this study, once the metabolic energy consumption was normalized for speed, the correlation improved 62.5 % for W_tot_, 4.3 % for W_ext_, and more than doubled for W_int_. This increase may be rooted in the relationship between energy recovery (i.e. energy transfer) and walking speed.

Optimized energy transfer during walking would be inversely related to metabolic cost [29]. The results of this are indicative of this notion. There was a significant positive relationship between the percentage of energy recovered and walking speed (Fig. 3). In addition, women in this study had walking speed significantly lower than that of reported optimal walking efficiency where energy recovery is the highest [10, 11, 30]. Furthermore, as energy transfer demonstrated significant inverse correlation with the measures of gross O_2_ cost (*r* = −0.44, *p* = 0.002) and net O_2_ cost (*r* = −.328, *p* = .023). These results are in agreement with previous findings of Willems et al. [27] and Olney et al. [25].

## Conclusion

Therefore, measures of mechanical work, when adjusted for resting energy expenditure and walking speed, may be useful in comparing metabolic energy consumption of gait between women during pregnancy, or longitudinal assessment of the same individual throughout pregnancy. Although mechanical work may not account for the variability in metabolic cost stemming from the foetal development, normalizing for REE and speed of walking, may allow mechanical work to predict the changes in gait during pregnancy.
